# Children’s Socioemotional Strengths in Early Childhood Education (ECE) and Before/After School Care (BASC): A Multilevel Ecological Analysis

**DOI:** 10.3390/children13010023

**Published:** 2025-12-23

**Authors:** Imogen M. Sloss, Nicola Maguire, Dillon T. Browne

**Affiliations:** 1 Department of Psychology, University of Waterloo, 200 University Ave. W., Waterloo, ON N2L 3G1, Canada; isloss@uwaterloo.ca; 2 The Learning Enrichment Foundation, 116 Industry St., Toronto, ON M6M 4L8, Canada

**Keywords:** socioemotional strengths, early childhood education, socioeconomic status, neighbourhood census, multilevel modelling

## Abstract

**Background/Objectives:** The current study explored how trajectories of children’s socioemotional strengths were explained by school, classroom, and individual differences in the context of licensed early childhood education (ECE), involving preschool and before/after school programming. The predictive role of neighbourhood socioeconomic status (SES) was also explored. **Methods:** Participants included *n* = 226 children from 39 classrooms across seven ECE centres in a large city in Canada. Educators completed measures of children’s socioemotional strengths at three time points between January and June 2024. Children’s forward sortation areas (FSA) were also linked with publicly available data on neighbourhood SES from the 2021 census. Four-level multilevel models estimated scores across time, individual, classroom, and school levels. **Results:** All four levels significantly explained variance in strengths. On average, child strengths improved over the 4.5 months of ECE programming. Random slopes at the individual and classroom level revealed variability in trajectories. Higher neighbourhood SES was associated with higher socioemotional strengths and was not associated with change over time. **Conclusions:** The findings of this study reveal that child, classroom, school, and neighbourhood factors interact to foster child socioemotional strengths. Thus, targeted and universal programs for promoting socioemotional development in ECE must similarly adopt a multiple levels of analysis perspective.

## 1. Introduction

Licensed early childhood education (ECE) and before-and-after-school care (BASC) are contexts that nurture children’s socioemotional strengths [1,2,3,4,5], where investments in early life yield the greatest lifetime return [6]. Licensed ECE and BASC providers are usually required to engage in empirical monitoring exercises, ensuring that children are growing in their socioemotional strengths, supporting accountability, while also promoting quality enhancement [7]. To date, there is a paucity of Canadian literature examining variation in children’s socioemotional strengths from a multilevel and ecological perspective, identifying important sources of variation not only across time but in addition to stable individual, classroom, and center-level differences. While such approaches have been applied in the US [1,8,9,10,11] and for academic outcomes [12], it remains an understudied question for ECE and BASC, especially within the unique Canadian context [13]. This information can be helpful in guiding policy and program planning investments. Therefore, the current study sought to explore trajectories of socioemotional strengths among children enrolled in licensed ECE (including preschool programs and BASC, as these are both part of ECE in Canada) with a major provider in a large Canadian city. A multilevel and ecological perspective was employed, whereby classroom and school-level differences in trajectories were investigated. Following perspectives highlighting the importance of social determinants of child and family well-being [14], the predictive role of census-linked socioeconomic status of children’s neighbourhood of residence was also explored.

### 1.1. Socioemotional Strengths

Socioemotional strengths begin to develop during infancy, and are associated with educational, occupational, physical, emotional, social, and behavioural outcomes across the lifespan [15,16]. Multiple aspects of children’s environments interact to foster or hinder the development of these strengths [1,4,17]. The Collaborative for Academic, Social, and Emotional Learning [18] explains that socioemotional strengths allow individuals to develop and work towards goals, persist in the face of setbacks, understand and regulate emotions, cope effectively with challenges, build secure and healthy relationships, and understand others’ perspectives. These strengths are often grouped into the following five key areas: decision making, self-management, self-awareness, social awareness, and relationship skills [18]. Researchers have found that socioemotional strengths play a significant role in myriad long-term outcomes, including life satisfaction, physical and mental health, socioeconomic status (SES), substance use, criminal activity, and social relationships [3,15,16,19]. Thus, exploring variation in socioemotional strengths across layers of the developmental ecosystem can help guide families, educators, service providers, and policy makers to nurture these strengths and their associated outcomes.

Various researchers have outlined the developmental systems that give rise to socioemotional strengths [20,21,22]. These strengths are the product of a complex interaction between a child’s temperament, genetics, and life experiences. Socioemotional strengths begin to develop as an infant forms an attachment with their primary caregiver. Over the first years of life, this growth expands to include the development of emotion regulation, self-control, attention, social skills, empathy, and autonomy [22]. As biological maturation propagates in a supportive social surrounding, the developmental ecology becomes more complex, and children’s strengths are further shaped by various contexts in a child’s life, including their family, neighbourhood, classroom, care providers, school, and peers. Furthermore, children’s experiences are informed by sociodemographic contexts, including SES, which are unequally distributed due to the concentration of resources within various groups in society [14]. Therefore, some groups tend to experience greater economically induced stress and limited access to development-enhancing resources and opportunities [14]. Empirical studies of children’s socioemotional strengths should measure and model these influences, given their demonstrable role in predicting outcomes. Additionally, these studies should operationalize socioemotional strengths in accordance with the sample’s developmental stage [23]. Therefore, the current study employed valid and developmentally sensitive measures of socioemotional strengths [24,25,26].

### 1.2. Contextual Influences on Socioemotional Strengths

#### 1.2.1. Socioeconomic Status (SES)

SES is a latent dimension comprising various economic indicators including an individual, family, or neighbourhood’s level of education, income, and access to economic capital (e.g., assets). Developmental researchers often focus on SES’ role in children’s growth and development, which remains an essential domain of research and policy. Indeed, researchers have found that children from lower-SES families have lower socioemotional strengths compared to children from higher-SES families [4,8,27]. Differences in these strengths are observable before children enter school [28]. Furthermore, the magnitude of this difference in socioemotional strengths gets wider as children get older [4].

There are many reasons why children from lower-SES families experience greater challenges to socioemotional skill development, including access to opportunity and psychosocial stress [28,29]. In general, lower-SES families have access to fewer resources compared to higher-SES families. Limited financial resources mean that lower-SES families may struggle to pay for educational materials and high-quality services or experiences for their children [4], impacting the type of stimulation that children receive. Additionally, neighbourhood factors can impact socioemotional strengths. In some jurisdictions, ECE centres and schools in lower-SES areas receive less funding, reducing the quality of these services [4,30,31]. A study by McCoy and colleagues [31] elucidated how these neighbourhood risk factors may impact development: they found that preschool program quality mediated the relationship between neighbourhood poverty and socioemotional strengths. In addition to difficulties accessing resources, lower-SES families and neighbourhoods tend to experience higher levels of stress and adversity. Masarik & Conger [32] outline how these stressors impact the family system and child well-being. Financial stress and community disadvantage increase caregiver stress, which can spillover into the marital and parent-child relationship [32]. When caregivers experience high levels of stress, they may have less capacity to be supportive and involved in the lives of their children [32]. Furthermore, caregivers experiencing high levels of stress may be less consistent in how they interact with their children [32].

#### 1.2.2. Early Childhood Education and Before/After School Care

Another prominent context in which socioemotional strengths develop is in ECE and BASC. For example, ECE and BASC environments allow children to interact with peers, follow routines, and comply with rules, providing opportunities to foster socioemotional development [1,17]. As a result, many researchers have explored ECE and BASC contexts in relation to socioemotional development. High-quality ECE and BASC programs are associated with many short- and long-term benefits, including socioemotional strengths [1,33]. For example, these high-quality programs are related to well-being, social skills, emotion regulation, inhibitory control, and academic achievement [3,13,34,35,36]. One pathway in which children develop these strengths is through play, as interacting and playing with peers fosters problem-solving, cooperation, perspective-taking, sharing, and self-restraint [37]. Furthermore, Jennings & Greenberg [21] and Jones & Bouffard [38] present models that outline how the classroom environment can foster socioemotional strengths. They posit that there are bidirectional relationships between educator socioemotional strengths and well-being, student–educator relationships, classroom management, classroom climate, and child socioemotional strengths, which is supported by research [8,39,40]. Moreover, these relationships are influenced by school and community contextual factors, highlighting the importance of considering a child’s developmental ecology [4,39,40].

ECE and BASC are demonstrably linked to socioemotional skill development in children. Yet, multilevel and ecological studies of children’s developmental health demonstrate systematic variation in outcomes as a function of the multiple and hierarchical levels of a child’s developmental system [14]. For example, research by Rasbash and colleagues (2010) suggests that multilevel contexts, including the individual, family, school, and neighbourhood level, each explain variance in children’s early educational trajectories, some of which is ostensibly linked to social disadvantage [12]. Notably, ECE appears to counteract some of the negative influence that low-SES environments have on socioemotional skill development, helping children from lower-SES households catch up to their peers [1]. In addition to short-term improvements, ECE programs are related to long-term socioemotional benefits. A meta-analysis of preschool programs for children from low-SES households found that they are associated with positive socioemotional functioning throughout school and into adulthood [10]. Furthermore, Reynolds et al. [11] reported the 19-year outcomes of children who participated in high-quality care versus those who participated in typical care. They found that high-quality care was associated with higher educational attainment and full-time employment, and lower rates of depressive symptoms, criminal activity, disability, and out-of-home placements [11]. At present, it remains unclear how children’s socioemotional outcomes in Canadian ECE and BASC vary across the levels of a child’s developmental ecology, as studies are often not designed to partition these relative sources of variance. This is problematic, as understanding the multiple sources of variability in socioemotional strengths, and their relative contributions, can help organizations and policy makers direct limited resources to enhancing child well-being.

### 1.3. The Present Study

The present study aimed to address these gaps by exploring the trajectories of educator-reported socioemotional strengths for children enrolled in ECE centres providing preschool and BASC programs in a Canadian context. The research questions investigated in this study include the following: (1) What proportion of variance in socioemotional strengths is attributed to (a) change over time, (b) child differences, (c) classroom differences, and (d) school differences? (2) How do socioemotional strengths change over 4.5 months of programming? (3) Does neighbourhood-level SES predict socioemotional strengths at baseline and over time?

We hypothesized that all four levels (change, child, classroom, and school) would explain a significant proportion of variance in strengths. Secondly, we predicted that strengths would improve over time. Finally, we expected that children from neighbourhoods with lower SES, as indexed through linkage with the Canadian census, would have lower strengths at baseline, and would improve more during the study period.

## 2. Materials and Methods

### 2.1. Participants and Procedure

Study participants were children enrolled in infant (*n* = 12), toddler (*n* = 32), preschool (*n* = 49), and kindergarten/school-aged programming (*n* = 133) at seven ECE centres run by a community agency in North-West Toronto, Ontario. These centres operate within public school settings and include full-day programs for preschool-aged children, as well as before-and-after school care (BASC) programming for school-aged children, which is a common practice in Canada. Keeping with the best-practice principles of community-based research [41], the agency was empowered to select priorities, including identifying the specific centers that they felt would be optimal in implementing this research protocol. All centres are licensed within the province of Ontario, Canada, and because of the federal government’s ECE program, families were able to access subsidies to lower the significant cost of care. The organization emailed caregivers with details about the study, asking if they would like their child to participate. Caregivers provided informed consent to have educators (registered early childhood educators and early childhood assistants) complete strengths-based measures about their children over three time points (January 2024, March 2024, and June 2024). Educators were trained to complete the paper surveys used in the study. They completed the surveys during paid hours when they had classroom coverage from other educators. Upon completion, the paper copies were transferred to the researchers, where research assistants inputted data into Qualtrics. The paper copies were stored in a secure location at the agency and university. The educators in the study were not considered to be research participants because they were engaging in tasks that were part of their routine job (filling out regular measures for children), and educator information was anonymized; therefore, educators did not need to provide consent. The current convenience sample included *n* = 226 children and ranged in age from 0.5 to 10 years (M = 4.60, SD = 2.21), which maps onto the age range of children served by the agency partner. Inclusion criteria required that the children were enrolled at one of the seven centres. There were no exclusion criteria for children within these centres. Children were identified as boys (*n* = 108), girls (*n* = 108), and gender minorities (*n* = 10). These children were from *n* = 39 classrooms in the seven ECE centres. There was variation in the number of children at each time point due to attendance changes (T1, *n* = 215; T2, *n* = 213; T3, *n* = 217), and all children were retained in analyses. Ethics clearance (#40760 and #43229) for this study was obtained by the IRB at [blinded].

### 2.2. Measures

#### 2.2.1. Outcome Variable: Strengths

**School-aged children.** The 72-item Devereux Student Strengths Assessment [DESSA; 24] was used to assess educator-reported child strengths for school-aged children (kindergarten through Grade 8, though the oldest children in the study were approximately Grade 4). Educators rated the frequency of all items during the past four weeks on a five-point Likert scale from “Never” to “Very Frequently”. Higher scores indicated higher levels of strengths. Sample items include: “offer to help somebody” and “work hard on projects”. Internal consistency was excellent (Cronbach’s alpha = 0.99).

**Preschoolers.** Strengths for preschoolers (children aged three to five years) were assessed using the 27-item Devereux Early Childhood Assessment for Preschoolers Second Edition [DECA-P2; 24]. Educators were instructed to rate the frequency of specific behaviours over the past four weeks on a five-point Likert scale from “Never” to “Very Frequently”. Higher scores on this measure indicated greater strengths. Example items are: “listen to or respect others” and “keep trying when unsuccessful”. This measure had good internal consistency (Cronbach’s alpha = 0.85).

**Toddlers.** The 36-item Devereux Early Childhood Assessment for Toddlers [DECA-T; 26] assessed strengths in children aged 18 to 36 months. Educators rated how frequently (five-point Likert scale from “Never” to “Very Frequently”) toddlers engaged in various behaviours. Higher scores reflected higher levels of strengths. Sample items include: “ask to do new things” and “handle frustration well”. The internal consistency of this measure was excellent (Cronbach’s alpha = 0.97).

**Infants.** Educators completed the 33-item Devereux Early Childhood Assessment for Infants [DECA-I; 26] to assess the strengths of infants between one to 18 months old. Educators rated the frequency of the items on a five-point Likert scale from “Never” to “Very Frequently”, with higher scores indicating higher strengths. Sample items include: “imitate actions of others” and “explore surroundings”. This measure had good internal consistency (Cronbach’s alpha = 0.92).

Raw scores for all measures of socioemotional strengths were converted to percentiles to contextualize the scores in reference to a normative sample. Although the use of percentile scores can pose limitations in growth models, the present study employed percentiles to allow for the comparison of children at various developmental stages.

#### 2.2.2. Predictor Variable: Neighbourhood Socioeconomic Status

In line with Jones and colleagues [42], neighbourhood-level socioeconomic status (SES) based on a child’s Forward Sortation Area (FSA; an alpha-numeric geo-code that describes large neighbourhoods throughout Canada) was taken from the Canadian 2021 Census [43]. An SES composite was created by considering the percentage of adults within an FSA who (1) were unemployed, (2) did not have a high school diploma, (3) were receiving government transfers, (4) led single-parent families, and (5) were living below the low-income line. Factor analysis revealed that these five variables loaded onto one factor, explaining 67.3% of the variance in the data. Higher scores reflected more socioeconomic disadvantage. The internal consistency was good (Cronbach’s alpha = 0.87).

#### 2.2.3. Covariates

Covariates included age (in years), gender, and program type. Dummy variables for program type included toddler (toddlers = 1, other = 0), preschooler (preschoolers = 1, other = 0), and school-aged (school-aged = 1, other = 0), which were all compared to infants (0 for all). Dummy variables for gender included girl (girls = 1, boys/gender minorities = 0) and gender minority (gender minorities = 1, girls/boys = 0), who were compared to boys (0 for both).

### 2.3. Statistical Analyses

Four-level multilevel modelling was conducted in RStudio (Version 2025.09.0+387) with the nlme package [44]. Repeated measures were nested in children, who were nested in classrooms, that were nested in schools. The models were compared with the likelihood ratio test and estimated with restricted maximum likelihood. Confidence intervals were created for the fixed and random effects using the profile likelihood method. Outliers for the study variables were winsorized. Assumptions were checked with Q-Q plots for the residuals at all 4 levels. A null model was created for RQ1 to determine the proportion of variance, using intraclass correlation coefficients (ICCs), in strengths due to the four levels of the multilevel model: change over time and error (level 1), child differences (level 2), classroom differences (level 3), and school differences (level 4). To explore the grand mean linear trajectories of strengths over time for RQ2, we added a fixed effect of time (months). Then, we added random intercepts and random slopes at levels 2, 3, and 4, which permitted individual children, classrooms, and schools to have different intercepts and trajectories. The random slope at level 4 was removed in our final model, because of unreasonably large confidence intervals, along with visual inspections of the histogram of residuals, revealing large departures from normality. Lastly, we added covariates and predictor variables as main effects for RQ3, to determine whether they predicted baseline strengths. Predictor variables were also added as an interaction with time to explore whether they predicted change.

#### Missing Data

Little’s MCAR test was conducted for each age group. The results were non-significant for infants (*X^2^*(219, *N* = 32) = 192.52, *p* = 0.901), toddlers (*X^2^*(777, *N* = 101) = 736.77, *p* = 0.847), and preschoolers (*X^2^*(786, *N* = 149) = 793.19, *p* = 0.422). However, Little’s MCAR test was significant (*X^2^*(4082, *N* = 399) = 4524.36, *p* < 0.001) for school-aged children, revealing that data were not missing at random. To deal with missing data, random forest multiple imputation was conducted with the missForest package [45]. This method uses a single imputation procedure, with evidence to suggest that it outperforms multiple imputation in terms of minimizing parameter bias associated with missingness [46,47].

## 3. Results

Descriptive statistics and bivariate correlations are presented in Table 1 and Table 2.

### 3.1. Question 1: Variance Partitioning

According to the ICCs, each level of the multilevel model, portrayed in Model 1 of Table 3, explained a significant proportion of child strengths in the null model: 32.7% of the variance was due to change (and potentially measurement error), 24.3% was due to child differences, 20.8% was due to classroom differences, and 22.2% was due to school differences.

### 3.2. Question 2: Growth Model

**Figure 1:**
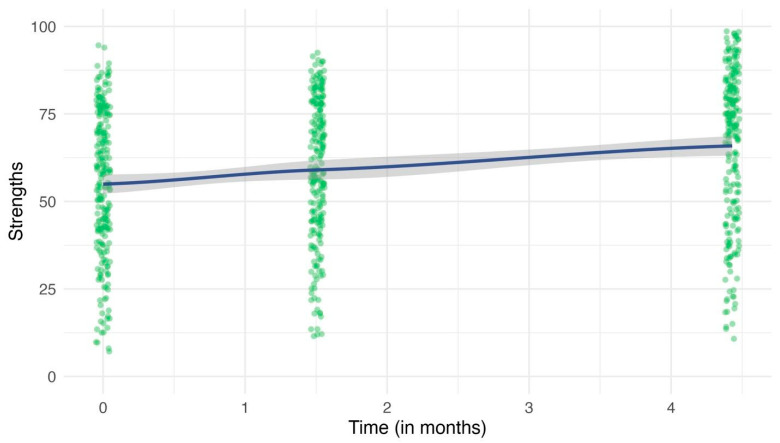
Trajectories of child strengths. The green dots represent individual participant values at each time. The blue line represents the mean trajectory for all participants over time. The grey shading represents *95% CIs*.

**Figure 2:**
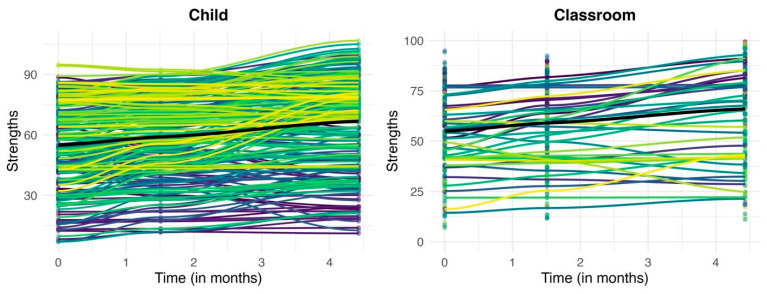
Child- & classroom-level trajectories of child strengths. Each coloured line represents the trajectories of an individual child (**graph on left**) and an individual classroom (**graph on right**). The black line represents the mean trajectory for all participants.

The growth model for child strengths is presented in Figure 1 and Models 2 and 3 in Table 3. On average, strengths improved over time (*b* = 2.69, 95% *CI* = 1.95, 3.42), improving approximately 2.69 percentile points each month. Significant random slopes (displayed in Figure 2) at level 2 (child level; *b* = 1.39, 95% *CI* = 0.83, 2.33), level 3 (classroom level; *b* = 3.54, 95% *CI* = 2.57, 4.88), and level 4 (school level; *b* = 1.44, 95% *CI* = 0.37, 5.65), revealed that individual children, classrooms, and schools had different slopes. In Model 3, the covariance was not significant at any level, suggesting that starting values of strengths did not affect rate of change. However, in Model 4, the child-level covariance was significant: children who started with lower strengths improved more over time compared to children with higher initial strengths

### 3.3. Question 3: Predictor Variables

**Figure 3:**
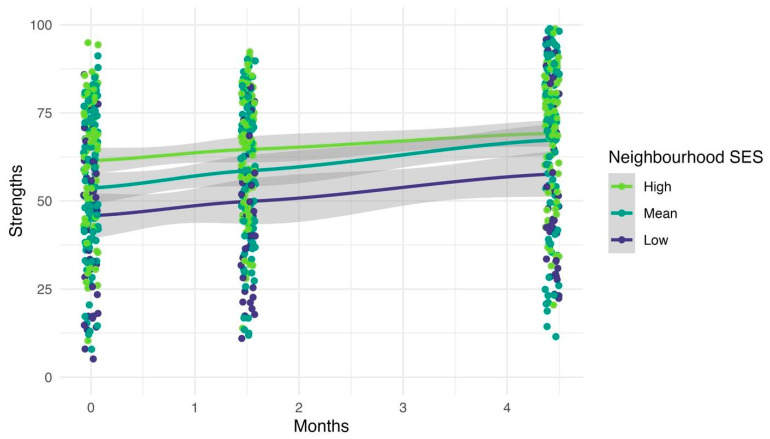
Average child strengths over time by neighbourhood socioeconomic status. Coloured lines represent strength trajectories based on levels of neighbourhood socioeconomic status (SES). The grey shading represents *95% CIs*.

Various predictor variables, displayed in Model 4 of Table 3, were significantly associated with child strengths at baseline and over time. Older age was associated with higher baseline strengths (*b* = 4.04, *95% CI* = 1.56, 6.52). Gender also significantly predicted strengths: girls had significantly higher strengths than boys at baseline (*b* = 9.77, *95% CI* = 5.09, 14.46). Gender minorities did not significantly differ from girls or boys. There were also significant differences in strengths by program: infants and toddlers had significantly higher strengths than preschoolers and school-aged children. Infants and toddlers, and preschoolers and school-aged children did not significantly differ from one another in strengths. Neighbourhood SES predicted strengths at baseline (*b* = −1.38, *95% CI* = −2.56, −0.19) and was not associated with change over time (see Figure 3). In other words, greater levels of socioeconomic disadvantage corresponded to lower socioemotional strengths, and this pattern was relatively stable over time. Stated differently, all children improved in their strengths equally, irrespective of the socioeconomic status of their neighbourhood. Neighbourhood differences are presented in the Supplementary Materials.

### 3.4. Sensitivity Analysis

To explore the role of developmental stage on child strength trajectories, interactions between program type (infant, toddler, preschool, school-aged) and months were added. None of these interactions were significant, suggesting that, on average, children improved at the same rate regardless of program type.

## 4. Discussion

The present study sought to investigate the trajectories of socioemotional strengths over 4.5 months of ECE or BASC enrollment, as well as the proportion of variance in strengths attributable to change, individuals, classrooms, and schools in a Canadian context. This research aimed to empirically monitor child trajectories to support accountability and promote quality enhancement for these centres [7]. Furthermore, children’s neighbourhood SES was included as an ecological predictor of baseline strengths and change. Our first hypothesis was supported: a significant proportion of socioemotional strengths was explained by all four levels, with change over time (plus measurement error) explaining the highest proportion of variance (32.7%; level 1), followed by child differences (24.3%; level 2), school differences (22.2%; level 4), and classroom differences (20.8%; level 3). Secondly, as expected, socioemotional strengths improved over time. In contrast to our hypothesis, neighbourhood SES predicted strengths at baseline, with children from higher-SES FSAs being reported to have higher strengths. However, neighbourhood SES was not related to change.

### 4.1. Predictors of Levels and Trajectories of Socioemotional Strengths

Change over time explained the greatest proportion of variance in socioemotional strengths, and linear trajectories suggested that child strengths improved over time, on average. Although our study did not employ a randomized control design, preventing us from concluding causation, it is possible that the ECE/BASC programs may have contributed to these observed improvements. This hypothesis would align with highly controlled research suggesting that high-quality ECE programs are associated with socioemotional strengths, in comparison to programs of lower quality educational supports [1]. Another possible mechanism of change is that as children aged, they developed more socioemotional strengths [22]. While measurements were age-normed and age was a control variable, the present study’s longitudinal design meant that time was elapsing while children completed the same set of tools (and norms), meaning that maturation effects are possible. In other words, the positive slope could be related to typical development, rather than the influence of ECE programming itself.

In the present study, the neighbourhood-level SES of children’s place of residence was associated with strengths, with children from lower-SES areas being reported to have lower baseline strengths. This finding is consistent with previous research, whereby low-SES is a risk factor for lower levels of socioemotional strengths [4,8,27]. Various mechanisms could explain why low-SES contexts may act as a risk factor in socioemotional development. Firstly, lower-SES areas tend to have fewer community resources due to levels of funding [4]. As a result, children may have fewer opportunities to access important services, and services that they do access may be less promotive of positive development [4]. Additionally, caregivers in these homes are at a greater likelihood of experiencing higher levels of financial- and community-related stressors, which could place strain on mental health and family functioning [4,32]. Consequently, caregivers may be less able to provide high levels of monitoring and consistency, both of which are related to socioemotional development [27]. Lastly, children from lower-SES neighbourhoods tend to be exposed to higher levels of adversity within the community v [4]. In response to these conditions, caregivers may be hesitant to allow their children to play outside, potentially reducing physical activity, an important predictor of socioemotional development [13,48,49].

Unfortunately, research suggests that the gap in socioemotional strengths between children from higher versus lower SES contexts widens as children get older [4]. However, in the current study, we found that, on average, all children improved at the same rate, regardless of neighbourhood SES. Although the children from lower-SES areas did not catch up to their peers during the study period–a replicated finding from previous ECE research [1,10,11]–it is possible that ECE programming prevented this gap from widening. Furthermore, many studies, including a 19-year longitudinal study by Reynolds and colleagues [11], explored long-term outcomes following ECE [1,10]. It is possible that our study was too short to capture the protective role of high-quality ECE for children from lower-SES areas. Nevertheless, our study was not a randomized control trial, preventing us from disentangling the impact of ECE versus maturation and other contexts in the children’s lives.

Although child strengths tended to improve over time, the significant random effects at the child, classroom, and school level revealed that children, classrooms, and schools displayed unique trajectories. Factors at these three levels of analysis will be explored below.

### 4.2. Socioemotional Strengths as an Individual-Level Phenomenon

The individual level explained the second largest proportion of variance in socioemotional strengths, suggesting that children systematically differ from one another in terms of strengths. Child age and gender were two individual-level variables that were found to predict socioemotional strengths. In the present study, age was positively associated with socioemotional strengths, suggesting that strengths increase as children get older, which replicates the findings of Hu et al. [8]. As mentioned above, children develop greater socioemotional strengths as they age, potentially contributing to this observed improvement. On average, girls had higher strengths compared to boys, which is consistent with previous research on socioemotional strengths [50]. Girls tend to be encouraged to express emotions, and reinforced for sensitive, compassionate and prosocial behaviour [51]. In contrast, boys may be encouraged to hide emotions other than anger, and to act in a more dominant and assertive way [51]. Given this approach to socialization, the American Psychological Association [52] has reported that exploring socioemotional development in boys and men is an important area for future inquiry. Family factors, which were not explored or isolated in the current study, may have also contributed to the child-level variance in strengths. Previous research has found that childhood adversity, parenting, and family functioning are associated with variation in socioemotional strengths [1,4,8,27]. Future studies might consider these family-level predictors of socioemotional strengths, given the substantial proportion of variance explained by the between-child level.

### 4.3. Socioemotional Strengths as a Classroom-Level Phenomenon

Jennings & Greenberg [21] and Jones & Bouffard [38] discuss the importance of teachers and schools in promoting socioemotional development. They assert that there are bidirectional relationships between a teacher’s own well-being and socioemotional strengths, student–teacher relationships, classroom management, classroom climate, socioemotional learning curriculum implementation, and student socioemotional strengths. Furthermore, they discuss how all these processes occur in the context of the school and community, highlighting the importance of considering the classroom ecology. In the current model, the classroom level explained the smallest—although still significant and sizable—proportion of variance in socioemotional strengths. This finding aligns with previous multilevel research in the context of ECE [8,9], including research in a Canadian context [13], and with theoretical models [21,38]. Unmeasured classroom- and educator-related variables may have contributed to this variance. Classroom organization, structure, and management are associated with socioemotional strengths [8,40]. Teaching techniques that involve small-group collaborative activities are associated with the development of socioemotional strengths [39]. In addition, classroom environments that foster a growth mindset are conducive to socioemotional development. For example, educators’ use of language (e.g., moving from “you can’t do it” to “you can’t do it yet”) and teaching strategies (e.g., providing students with the opportunity to redo work) can foster socioemotional strengths, such as positive coping, persistence, and goal-directed behaviour [39]. Student–educator relationships also play a role in positive socioemotional outcomes [39,40]; therefore, educator turnover negatively impacts socioemotional development [39]. Lastly, educators who have higher socioemotional strengths, and who experience lower levels of stress and mental health difficulties tend to be more sensitive to children’s mental and emotional states, and, therefore, have a greater ability to foster these strengths in students [39,40,53]. Educators should strive to implement these classroom management strategies and teaching styles, as well as personal self-care strategies, to ensure that their classroom has the potential to foster socioemotional strengths.

### 4.4. Socioemotional Strengths as a School-Level Phenomenon

The results revealed that the school level explained a significant proportion of variance in child strengths. These findings differ somewhat from previous multilevel modelling studies, whereby the school level did not significantly contribute to variance in socioemotional strengths in ECE settings [8,9]. However, when exploring other outcomes, such as academic achievement, multilevel modelling studies have identified the school level as a significant source of variability [54,55]. Although not explored in the current study, school characteristics may contribute to the variance observed at this level. School funding has implications for student socioemotional development. For example, schools with more funding tend to have more educational resources, lower student–educator ratios, and the ability to hire the most experienced educators [4]. Furthermore, counselling and emotional support services are sometimes available to support youth, fostering their socioemotional strengths [39]. In addition, schools with more funding may be able to provide socioemotional strengths training, explicitly equipping educators to foster these strengths [39,40]. School culture can also play a role in student strengths. For example, schools that are committed to fostering equity and inclusivity may implement programs that support students from underrepresented groups [39]. Furthermore, support between staff members is key to a school climate that fosters socioemotional development [39,40]. Lastly, relational discipline strategies that support students in repairing harm and learning from their behaviour can promote socioemotional strengths [39]. Future Canadian ECE research that explicitly measures these school-level (and classroom-level) factors will be informative for service providers, organizations, and policy makers in planning programming and educator development aimed at growing children’s socioemotional well-being.

### 4.5. Implications

There are several implications that emerge from this multilevel study of children’s ECE and BASC. First, the comparable rate of growth in socioemotional strengths between children from lower- and higher-SES communities suggests that ECE and BASC programs might promote socioemotional development among families who are facing economic uncertainty. These findings highlight the importance of expanding low-cost, high-quality ECE programs for lower-SES families, and are informative considering Canadian national programs to make ECE and BASC more affordable for families. However, future longer-term studies should be conducted to further explore the relationship between SES and strengths in a Canadian context. The result that the highest proportion of variance was explained by change over time suggests that socioemotional strengths do change during infancy, toddler years, and childhood. Therefore, families, educators, and policy makers should capitalize on this formative period by seeking to promote environmental predictors of child strengths at the individual, classroom, school, and neighbourhood level. The Heckman Equation (n.d.) provides recommendations for fostering positive lifelong skills. Supporting socioemotional development should begin at birth, and ECE programs for infants as young as eight weeks old are associated with greater school readiness. Furthermore, they emphasize the importance of continuous care, as this provides consistency in relationships, a key component in the formation of secure attachments. In addition, they assert the critical role that parents play in child development, and advise that ECE programs involve parents (e.g., psychoeducation), given that the family environment plays a significant role in socioemotional strengths. Next, they recommend that educators and teachers receive training to develop their own socioemotional strengths and learn how to interact with students in ways that promote positive development. Lastly, the Heckman Equation (n.d.) emphasizes the importance of considering skills across domains (e.g., socioemotional strengths, cognitive ability, academic skills), as these areas are interrelated, and influence each other throughout development. ECE providers should strive to implement these recommendations, given the significant role that the ECE context plays in child development.

### 4.6. Strengths, Limitations, and Future Directions

The present study had several strengths. The proportion of variance explained by multilevel contexts could be identified due to the hierarchical and longitudinal nature of the study data. Additionally, this research adds to the literature about ECE and BASC in a Canadian setting. Another key strength of this study is that information from the Canadian census could be linked to individual participants, allowing for the exploration of neighbourhood-level SES disadvantage on trajectories of strengths.

The current study also had various limitations. Firstly, all measures were completed by educators, which may have inflated the classroom-level variance. In addition, educator ratings may not apply to a child’s strengths in contexts outside of ECE. Future studies could include ratings from multiple informants or an observational design. Future research would also benefit from classroom- and school-level predictor variables. Next, our predictor variables explored SES at the neighbourhood level as opposed to the family or household level. While neighbourhood SES that is directly linked from the census is a robust and commonly used metric, it is also true that there is within-neighbourhood variation in SES as well. Therefore, a strength of the current study is that we were able to explore the role of a child’s neighbourhood in their socioemotional development. However, we did not have detailed family-level information, which may have further clarified the relationship between SES and socioemotional strengths. Additionally, while we explored the proportion of variance explained by school and classroom differences, we did not include any predictors at these levels, preventing us from understanding how these environments affect socioemotional strengths. Furthermore, the measures assessed different strengths across developmental stages, complicating the interpretability of results; however, the moderation analysis aimed to explore developmental differences in trajectories (see sensitivity analyses). Next, our use of percentiles instead of raw scores in our growth models was a limitation, as the trajectory of the statistically average child would be flat. Yet, the proximity of repeated measures allowed us to isolate and identify growth in strengths within a relatively short developmental period (i.e., less than half a year). In addition, all children were enrolled at various centres run by the same organization, which may limit generalizability beyond this organization. Selection bias may also have influenced the results, as there may have been systematic differences between families who did and did not consent to participate in the study. Lastly, we cannot conclude whether ECE participation caused the observed improvements in strengths because the current study did not employ a randomized controlled trial design. Future studies should include random assignment and a control group to disentangle the influence of ECE from other multilevel predictors of socioemotional development.

## 5. Conclusions

The current study aimed to investigate trajectories of children’s socioemotional strengths in the context of ECE, along with the role that contextual factors across multiple levels of analysis play in the development of these strengths. The results revealed that a significant proportion of variance in strengths was attributed to school, classroom, child, and time differences (RQ1). On average, children improved in socioemotional strengths over the 4.5 months; although, some schools, classrooms, and children improved at a faster rate than others (RQ2). Lastly, lower neighbourhood SES was associated with lower strengths at baseline but was not associated with change over time (RQ3). The findings from the current study should be interpreted with caution, as all participants were from centres within the same organization. The findings of the current study emphasize the importance of comprehensively considering a child’s multilevel context to foster positive socioemotional development. The relatively equal weight across temporal, individual, classroom, and school differences speak to the balancing of universal and targeted strategies when designing programming and policy aimed at promoting children’s socioemotional strengths.

## Figures and Tables

**Table 1:** Descriptive statistics and bivariate correlations between study variables.

Variable	2	3	4	5	*M*	*SD*
1. Age	−0.21 *	0.11 *	0.08 *	−0.01	4.60	2.21
2. SES disadvantage	–	−0.25 *	−0.14 *	−0.17 *	15.32	4.42
3. Total Strengths T1		–	0.56 *	0.53 *	52.65	27.61
4. Total Strengths T2			–	0.64 *	62.49	26.43
5. Total Strengths T3				–	65.66	27.07

*Note. M* = mean/average; *SD* = standard deviation; SES = socioeconomic status. * denotes significance at the *p* < 0.05 level.

**Table 2:** Mean strengths by gender, timepoint, and program.

	Boys (0)		Girls (1)	
	*M*	*SD*	*M*	*SD*
Time 1				
Infants	–*	–*	66.46	19.53
Toddlers	46	33.74	47.75	30.45
Preschoolers	42.81	31.84	52.87	28.56
School	50.38	26.83	60.97	23.11
Time 2				
Infants	–*	–*	76.92	12.49
Toddlers	55.32	36.65	66.15	33.63
Preschoolers	54.38	28.91	63.45	26.32
School	58.11	26.86	67.8	20.68
Time 3				
Infants	–*	–*	–*	–*
Toddlers	64.68	34.11	80.95	19.59
Preschoolers	61.96	35.86	65	23.79
School	56.81	28.91	70.47	18.97

*Note. M* = mean/average; *SD* = standard deviation. Gender minorities were suppressed due to cell size. –* were suppressed due to cell size.

**Table 3:** Fixed and random effects of the multilevel model output examining school, classroom, and child trajectories on child socioemotional strengths.

	Model 1	Model 2	Model 3	Model 4
Fixed Effects	*b [95% CI]*	*b [95% CI]*	*b [95% CI]*	*b [95% CI]*
Intercept	54.86 * [44.17, 65.55]	49.54 * [38.75, 60.33]	50.58 * [41.41, 59.76]	67.04 * [50.46, 83.62]
Months		2.69 * [1.95, 3.42]	2.16 * [0.43, 3.90]	2.45 * [1.04, 3.85]
Age				4.04 * [1.56, 6.52]
Toddler				−5.15 [−16.37, 6.07]
Preschool				−24.73 * [−40.28, −9.18]
School				−24.00 * [−42.45, −5.55]
Girl				9.77 * [5.09, 14.46]
Gender minority				5.52 [−5.83, 16.86]
SES disadvantage				−1.38 * [−2.56, −0.19]
Months * SES disadvantage				0.02 [−0.23, 0.27]
Random Effects	*SD [95% CI]*	*SD [95% CI]*	*SD [95% CI]*	*SD [95% CI]*
Level 4				
Random intercept	12.77 * [6.00, 27.18]	12.77 * [6.00, 27.18]	10.37 * [4.39, 24.51]	10.32 * [4.36, 24.41]
Random slope			1.44 * [0.37, 5.65]	
Cov (Intercept/Months)			0.79 [−0.95, 1.00]	
Level 3				
Random intercept	11.97 * [8.41, 17.04]	11.97 * [8.41, 17.03]	11.03 * [7.21, 16.89]	10.40 * [6.64, 16.28]
Random slope			3.54 * [2.57, 4.88]	3.68 * [2.71, 4.99]
Cov (Intercept/Months)			−0.20 [−0.60, 0.28]	−0.23 [−0.65, 0.30]
Level 2				
Random intercept	13.95 * [11.81, 16.47]	14.37 * [12.30, 16.79]	17.98 * [15.50, 20.86]	17.13 * [14.69, 19.98]
Random slope			1.39 * [0.83, 2.33]	1.57 * [0.98, 2.52]
Cov (Intercept/Months)			−0.98 [−1.00, 0.31]	−0.98 * [−1.00, −0.25]
Level 1				
Random intercept	18.80 * [17.62, 20.07]	17.82 * [16.70, 19.03]	15.36 * [14.35, 16.44]	15.30 * [14.29, 16.38]

*Note. b* = unstandardized estimates; *SD* = standard deviation; *95% CI* = 95% confidence intervals. * denotes significant findings based on the *95% CIs*.

## Data Availability

The dataset is not available to the public.

## Supplementary Materials

The following supporting information can be downloaded at: https://www.mdpi.com/article/10.3390/children13010023/s1, Figure S1: Total strengths for children enrolled in early childhood education (ECE) between January and April 2024, and census-linked socioeconomic disadvantage by neighbourhood. *Caption*: Neighbourhoods are defined by forward sortation area (FSA), which are the first three characters of a postal code. SES disadvantage was converted to *z*-scores.
